# St. Luke’s Hospital

**Published:** 1872-09

**Authors:** John E. Owens

**Affiliations:** Surgeon to the Hospital


A MONTHLY RECORD OF

*Medicine, Surgery and the Collateral Sciences.*

EDITED BY J. ADAMS ALLEN, M.D., LL.D.; AND WALTER HAY, M.D.

VOL. XXIX. — SEPTEMBER, 1872. — NO. 9.

### Original Communications.

ARTICLE I. — *St. Luke's Hospital. Ovariotomy—Perforation of Intestines—Death.* Under the care of JOHN E. OWENS, M.D., Surgeon to the Hospital.

M. A. G., aged 28, was admitted to St. Luke's Hospital, April 17th, 1872. She had been married nine years, had never borne children, but had considered herself a healthy woman till two years ago, when suddenly a severe pain, which subsided in a week, attacked her in the right side, just below the hepatic region. Previous to the attack of pain, she noticed that the belly was enlarging, and that her clothes had become too tight. The enlargement grew more and more prominent, until she was tapped, and four gallons and three pints of brownish fluid were drawn off. A few days after the tapping (which was performed in St. Louis), the patient felt a hard lump in the cavity of the abdomen. Defecation, urination and menstruation were normal. The appetite was good, and the patient slept well. She had a good recovery from the tapping. During the first two weeks, subsequent to tapping, she filled very rapidly. This was the patient's history of her case.

The urine was examined between April 17th and 24th, with the following result: Sp. gr. 1030, normally acid, excess of urates, excess of mucus. Six and a half gallons of ascitic fluid were withdrawn May 17th. The fluid was straw-colored, sp. gr. 1024, reaction slightly alkaline, albuminous, coagulating almost solidly. The tumor was considered by all who examined the case, and by myself, sufficiently movable to justify ovariotomy, which was performed August 2nd in the presence of Drs. Gunn, Byford, Jones, Hay, Powell, and others. Upon opening the abdomen, the tumor was found to be quite movable, there being but few adhesions about its upper part. The difficulties of removal were, however, very great on account of the existence of intimate and strong adhesion about the pedicle and base of the cysts. A small cancerous growth was found growing from the posterior wall of the body of the uterus. These adhesions being, for the most part, confined to the base, permitted a tolerably free movement of the tumor. The clamp used in this case was Green's modification of an instrument, devised by Hill, of Augusta, Maine, for the removal of intro-uterine polypi. Hill's instrument consists of a "steel rod three-sixteenths of an inch in diameter, with a perforated shoulder turned at one end, the other being flattened and bent into the spring;" the straight shaft is six and one-half inches long. The ligature, for which Dr. Hill employs hempen thread and annealed iron wire twisted together, is cast around the neck of the polypus; "the two ends are then passed through the shoulder, which is pushed firmly up to the pedicle, and fastened to the extremity of the spring while it is closed by the hand. The moment the hand relaxes its grasp, the force of the spring strangulates the growth, and this force is constantly maintained until the ligature cuts its way through."

It must be well known that a ligature applied in the ordinary way, ceases to constrict the pedicle as soon as it cuts its way and becomes imbedded in the tissues around which it may be applied. In 1867, I removed a solid tumor from the cavity of the abdomen; the ligatures applied in the usual manner did not ulcerate their way through the pedicle for nearly four weeks. This defect is entirely obviated by the employment of the instrument above described. Wm. Warren Green, M.D., professor of surgery in the Medical School of Maine, has modified Dr. Hill's instrument and applied it to the ligation of the pedicle of ovarian tumors. Green's modification consists of "a short steel shaft, moderately curved near the end, and grooved and perforated for the ligature. This screws into a steel plate, which supports upon its upper surface a short, upright, hollow cylinder, the tube being continuous with the groove of the shaft when fitted. An angular offshoot from this receives the spring. A screw and clamp are used for closing and holding the spring while the ligature is being adjusted. The ligature, which should be metallic (platinum being the best), being cast around the pedicle, its two ends are carried through the canal, and fastened while the spring is shut. The shaft rests in the lower angle of the incision, which is elsewhere closed. The plate rests upon the integument, the spring lying upon the pubis in the median line, and is supported laterally by the compress and binder properly adjusted."

I have described the "spring ligator" in Dr. Green's own words. A complete description by him may be found in "The Boston Medical and Surgical Journal," March 2nd, 1871.

The operation having been finished, the patient was placed in bed, warmth applied to the extremities, and stimulus administered. This patient operated upon Aug. 2nd, at 8:30 A. M., died August 20th at 1 P. M., of exhaustion from peritonitis and perforation of the small intestine, the result of inflammatory softening. The vomiting of a more or less acid greenish fluid began soon after the patient was placed in bed and continued with great obstinacy till the day of her death. Counter-irritation and ice-bags to the epigastrium, ices, sherry and ice, lime-water in various combinations, aromatic spirits of ammonia, bicarbonate of soda, and bismuth, being used as occasion seemed to require, produced little or no effect, except the bismuth, which, when given in forty-grain doses, rendered the vomiting somewhat less frequent. Opium in any form, as was found before the operation, was not tolerated, but chloral hydrate, taken immediately after vomiting, was the means of securing many moments of sleep. Nourishment consisted of broths, milk, sherry and egg, albumen with stimulants, administered both by the mouth and by the rectum. Catheterism was for a time employed, but eventually it became unnecessary.

August 5th. The wound except at its lower extremity had well closed by adhesive inflammation; diarrhœa during the day; pulse

120. Nausea was not marked, but the patient complained of constant burning at the pit of the stomach, and finally of the throat.

August 8th. Discharge from the abdomen began to grow offensive. From this date to within a day or two of her death, the cavity of the abdomen was washed out with carbolic acid water (strength 1 to 100 at first, and finally 1 to 80 of the temperature of the body), from one to three times daily, using at each washing from forty to one hundred and twenty ounces. Nothing answers the purpose of washing the cavity of the abdomen better than the ordinary nasal douche. A gum catheter attached to the free end of the rubber tubing is inserted into the abdominal cavity through the lower end of the wound. The glass having been filled, is raised above the patient's level, when the contents of the glass will readily find its way into the abdomen, after which, when the glass is depressed below the patient's level, the washings will rise in the glass, which may be emptied and if necessary refilled.

The night of August 9th, seven days after the operation, the wire ligature having cut its way through the pedicle, the "ligator" was removed.

August 10. Removed one suture pin, four silver wire sutures, and applied adhesive straps to support the wound.

August 11th. Remaining pins and wires removed.

August 13th. The discharge having been very profuse for some days, assumed at this date the character of fæces.

August 18th. The discharge saturated not only the body clothing, but the bed and bed clothes. With the hope of lessening the discharge of fecal matter from the wound, warm water enema was ordered. The greater part of the injection passed through the abdominal opening.

August 20th. Died at 1 o'clock, P. M. The pulse reached 132, and never fell below 120.

I. N. Danforth, M.D., pathologist to St. Luke's Hospital, kindly furnished to me the following report of the autopsy:

M. A. G., sectio cadaveris, 20 hours, post mortem. Body fairly nourished; rigor mortis strongly marked.

*Head* not examined.

*Chest.* Lung tissue healthy; lungs somewhat compressed and pushed up by the encroachment of abdominal organs; heart normal, except in regard to position, its apex pointing to the third intercostal space directly above the nipple.

*Abdomen.* (The dissection and investigation of the condition of the abdominal organs was much embarrassed by inflammatory adhesions, which were very general, and quite firm.) The liver was firmly adherent to the diaphragm throughout its whole convex surface, by old adhesions, and enlarged to about one-third more than its normal volume. Its convex surface was far more convex than usual, while its concave, or lower, surface was less concave or more flattened than usual; in other words, the hypertrophy had taken place, for the most part, in an upward direction, because it encountered less resistance in this direction—the distended condition of the abdomen below being sufficient to prevent any growth downward. The liver presented no evidence of disease of its parenchyma. The spleen was considerably enlarged (to the bulk of the kidney) and very much softened; in fact, it was a mere mass of black semi-liquid pulp; microscopic examination demonstrated that this pulpy mass for the most part consisted of broken-down blood corpuscles. I also find in the spleen many globular, white, shining bodies, of the size of a medium-sized shot—some of them exceedingly hard, some semi-solid. Chemical and microscopic examination proved them to consist of little cysts, filled with carbonate of lime. Undoubtedly they were the malpighian bodies, which had undergone calcareous degeneration. I find no parallel case recorded in works to which I have access, however. Considerable inflammatory engorgement was found in the stomach; its vessels were distended and tortuous. Pancreas not notably changed. The omentum was pretty firmly adherent to the intestines by recent adhesions. The coils of intestines were closely matted together by organized lymph, and their separation was tedious and difficult. A fistulous opening, about 1½ inches in length was found in the ilium about 12 inches above the ileo-cœcal valve; this was evidently the result of inflammatory softening. At this point the mucous lining of the canal was dark and cloudy with inflammatory engorgement; the membrane was also much softened. The fistulous opening was in the right iliac region. In the left side, extending from the tenth rib to the ant. sup. sp. process, in front of and covering the descending colon, was an abscess, holding not less than eight ounces of pus—its internal wall being formed by coils of intestine, and its anterior wall both by coils of intestine and the abdomen. The abdominal incision was quite firmly united throughout nearly its whole extent. Kidneys quite healthy.

*Pelvis.* The pelvis proved to be a sort of pathological museum. If possible, the pelvic organs were more perfectly fused together than those of the abdomen. The uterus was slightly enlarged, the cervix filled with tough, reddish mucus. From its posterior superior aspect, a little to the right of the median line, a cauliflower growth was found as large as a hen's egg; this proved to be a beautiful specimen of villous cancer. The right ovary could not be found, but the pedicle of the tumor, and some condensed connective tissue occupied its place. In place of the left ovary, I found a cyst, filled with fluid fat, and beautiful plates of cholesterine—evidently the commencement of a *second* ovarian tumor. Behind the uterus, and between it and the rectum, rising a little above the brim of the pelvis, was a large cyst filled with reddish or brownish serum. In this serum many corpuscles very like pus cells were found, and also many half-grown epithelial cells welded together in patches like pavement epithelium. Several smaller cysts were found also filled with the same brownish-red serum, except one which was filled with the same cauliflower growth as I have already mentioned. Considerable free pus of recent origin was found in the pelvis—the product of degenerated inflammatory exudation.

In addition to all this, I have to note the great change which the general connective tissue of the pelvis and its contained organs had undergone. The ligaments and peritoneal folds were thickened, indurated and dark colored. The areolar tissue had lost its delicate appearance and become thick, dense and tough.

It is rarely the case that so much pathology and so little physiology—using the latter term in its broader-gauged sense—is found in the human body.

